# Multivessel Coronary Vasospasm Causing Cardiogenic Shock After Anatomically Remote Pulsed Field Ablation for Atrial Fibrillation

**DOI:** 10.1016/j.jaccas.2026.108972

**Published:** 2026-06-20

**Authors:** Kanto Saida, Junichiro Miyamoto, Yu Michiura, Riki Nagatomo, Kenji Nagashio, Takunori Aoyama, Hitoshi Hirano, Akira Takayama, Noboru Ichihara

**Affiliations:** Department of Cardiology, Tokyo Metropolitan Bokutoh Hospital, Tokyo, Japan

**Keywords:** cardiogenic shock, dexmedetomidine, intracoronary nitroglycerin, posterior wall isolation, pulmonary vein isolation, vasospastic angina

## Abstract

**Background:**

Coronary vasospasm is an uncommon but serious complication after pulsed field ablation (PFA). Localized spasm is recognized near coronary-adjacent lesions, but generalized multivessel vasospasm after anatomically remote left atrial PFA is less defined.

**Case Summary:**

A 68-year-old man with persistent atrial fibrillation underwent pulmonary vein and posterior wall isolation with a 31-mm pentaspline PFA catheter. Immediately postprocedure, refractory hypotension and evolving ischemic electrocardiographic changes developed, initially in the inferior leads and subsequently in the anterior leads. Emergency coronary angiography revealed diffuse bilateral coronary vasospasm remote from treated sites on fluoroscopic and computed tomography/FARAVIEW assessment. Vasospasm rapidly resolved after intracoronary nitroglycerin, followed by continuous nicorandil and hemodynamic recovery.

**Discussion:**

Anatomical remoteness, dynamic electrocardiographic evolution, and nitrate reversibility supported generalized vasospastic reactivity rather than focal coronary injury. Autonomic, endothelial, hemolytic, and anesthetic factors may have contributed.

**Take-Home Messages:**

Severe but reversible coronary vasospasm can occur after anatomically remote left atrial PFA. When hypotension or evolving ischemic ST-segment changes occur after PFA, immediate coronary angiography and intracoronary vasodilator treatment should be considered.

## Visual Summary

**Figure:**
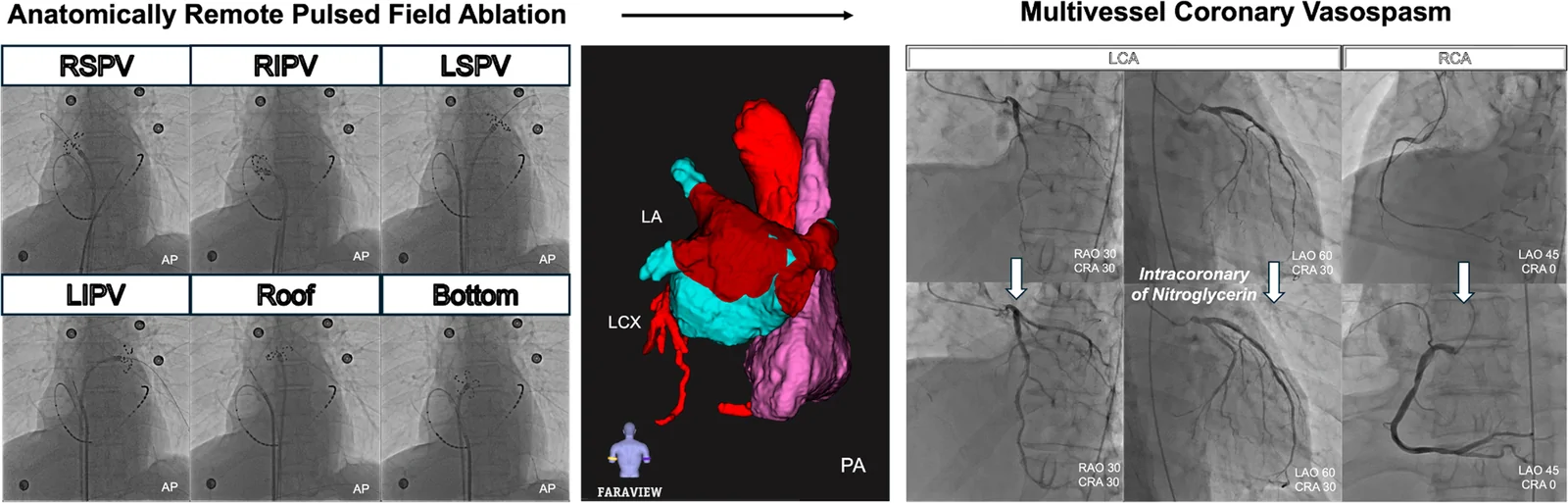

**Visual Summary:**
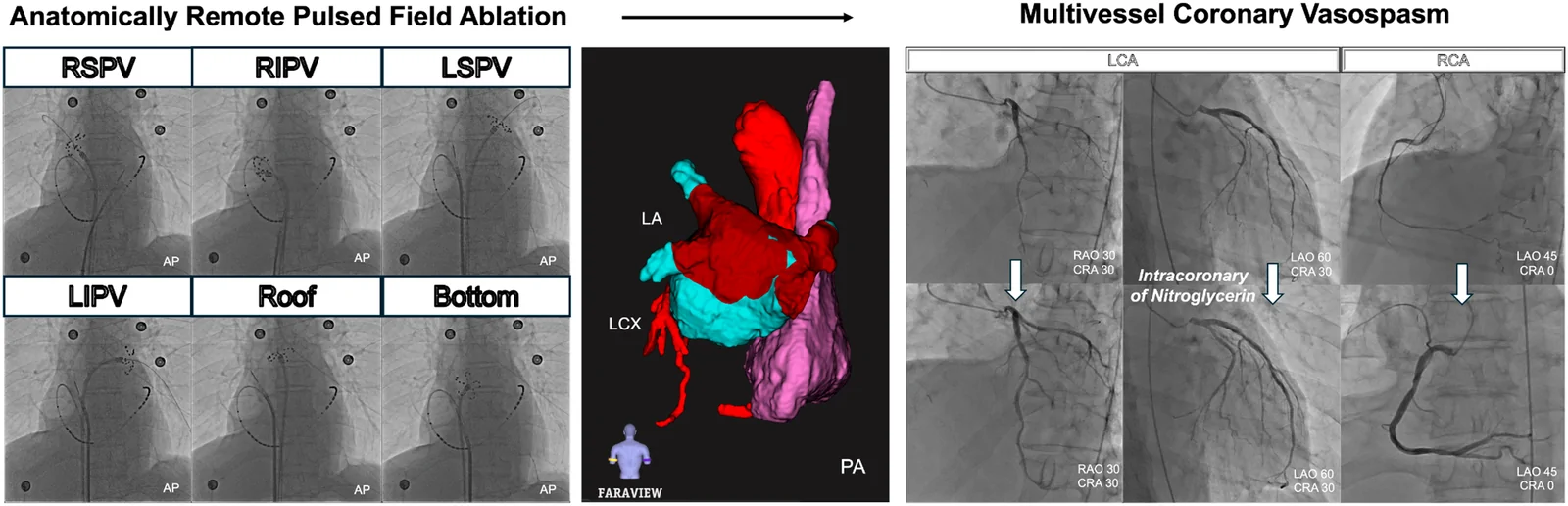
Remote PFA-Associated Multivessel Coronary Vasospasm The visual summary integrates the fluoroscopic PFA application sites, CT/FARAVIEW anatomic reconstruction, and coronary angiographic findings. Labels indicate the left atrium (LA), left coronary artery (LCA), left anterior descending artery (LAD), left circumflex artery (LCX), and right coronary artery (RCA). PFA was delivered for pulmonary vein isolation and posterior wall isolation at sites anatomically remote from the coronary arteries. After PFA, emergency coronary angiography demonstrated diffuse multivessel coronary vasospasm, which rapidly improved after intracoronary nitroglycerin. The composite supports generalized vasospastic reactivity rather than direct local coronary injury. CRA = cranial; CT = computed tomography; LAO = left anterior oblique; LIPV = left inferior pulmonary vein; LSPV = left superior pulmonary vein; PFA = pulsed field ablation; RIPV = right inferior pulmonary vein; RSPV = right superior pulmonary vein.

## History of Presentation

A 68-year-old man was admitted for catheter ablation of persistent atrial fibrillation after progressive exertional dyspnea. He had been followed for congestive heart failure with reduced left ventricular systolic function. Immediately after left atrial pulsed field ablation (PFA), he developed persistent refractory hypotension despite norepinephrine support, and monitor electrocardiography (ECG) demonstrated ST-segment elevation in leads II, III, and aVF.

**Take-Home Messages**

• Severe but reversible coronary vasospasm can occur after anatomically remote left atrial PFA.
• When hypotension or evolving ischemic ST-segment changes occur after PFA, immediate coronary angiography and intracoronary vasodilator treatment should be considered.

## Past Medical History

His past history included right lower lobe lung cancer treated with segmentectomy, dyslipidemia, peripheral artery disease treated with right superficial femoral artery intervention, and prior cerebral infarction. He had also been noted to have left ventricular thrombus and later left atrial appendage thrombus, both of which resolved after anticoagulation management.

## Differential Diagnosis

The immediate differential diagnosis included acute coronary syndrome, coronary vasospasm, coronary embolism, air embolism, pericardial tamponade, and sedation-related hemodynamic collapse.

## Investigations

Baseline 12-lead ECG showed atrial fibrillation without ischemic St-T changes (Figure 1). Transthoracic echocardiography demonstrated a left ventricular ejection fraction of 39% without regional wall motion abnormality. A prior coronary angiogram had shown focal stenosis in segment #9 without other significant obstructive disease. After the postprocedural onset of hypotension and ST-segment elevation, emergency coronary angiography demonstrated diffuse vasospasm involving both left and right coronary arteries (Figure 2). The angiographic narrowing rapidly improved after intracoronary nitroglycerin, supporting a reversible vasospastic mechanism rather than fixed coronary occlusion.

**Figure 1:**
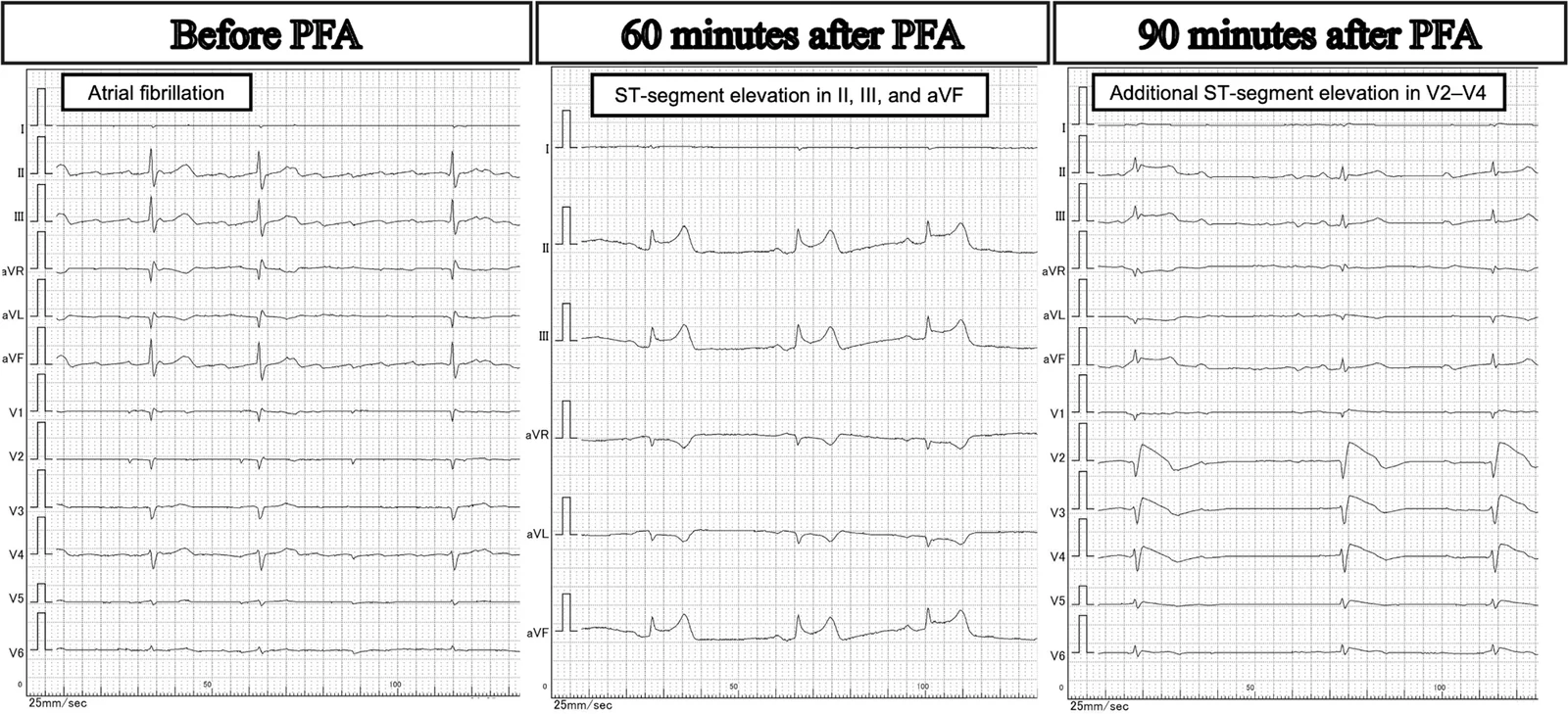
ECG Changes Before and After PFA Before PFA, the patient was in atrial fibrillation without significant ST-segment changes. At 60 minutes after the procedure, ST-segment elevation was observed in leads II, III, and aVF. Thirty minutes later, the ST-segment elevation in the inferior leads had improved, whereas additional ST-segment elevation was noted in leads V_2_-V_4_. The annotation boxes summarize the rhythm and key ST-segment findings at each time point. ECG = electrocardiography; PFA = pulsed field ablation.

**Figure 2:**
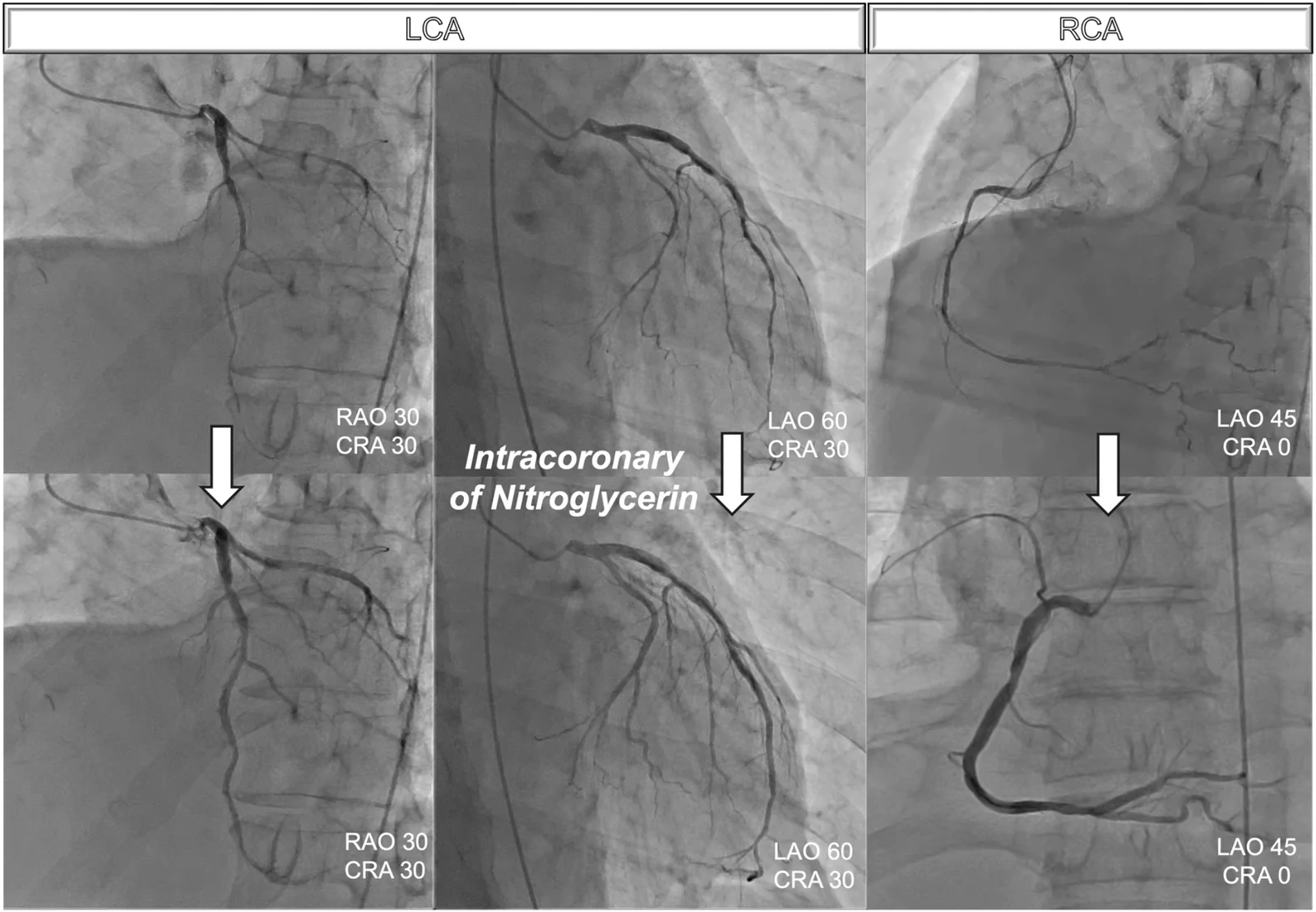
Coronary Angiographic Findings After PFA The upper panels show coronary angiography performed after PFA. Coronary vasospasm was observed in both coronary systems, with severe stenosis in the left anterior descending, left circumflex, and right coronary arteries. The lower panels show angiographic images obtained after intracoronary nitroglycerin administration. After nitroglycerin injection, coronary dilatation was achieved and the stenoses improved, consistent with vasospastic angina. CRA = cranial; LAO = left anterior oblique; LCA = left coronary artery; PFA = pulsed field ablation; RAO = right anterior oblique; RCA = right coronary artery.

## Management

The procedure was performed under deep sedation with dexmedetomidine and intermittent propofol, with invasive arterial pressure monitoring, noninvasive positive pressure ventilation, norepinephrine support, and systemic heparinization. PFA was delivered using a 31-mm pentaspline PFA catheter (FARAPULSE; Boston Scientific) for pulmonary vein isolation (PVI) and posterior wall isolation (PWI), with a total of 63 applications. Postprocedural fluoroscopic views were compared with coronary angiographic views and suggested that the ablation lesions were not adjacent to the coronary arteries (Figure 3). Computed tomography (CT)/FARAVIEW reconstruction was also used to assess the spatial relationship between PFA sites and coronary arteries (Figure 4). Because the patient developed refractory hypotension and inferior ST-segment elevation after the procedure, emergent coronary angiography was performed. Intracoronary nitroglycerin immediately relieved the coronary vasospasm. Continuous nicorandil infusion was then initiated, blood pressure gradually improved, norepinephrine was discontinued, and oral nicorandil was subsequently introduced.

**Figure 3:**
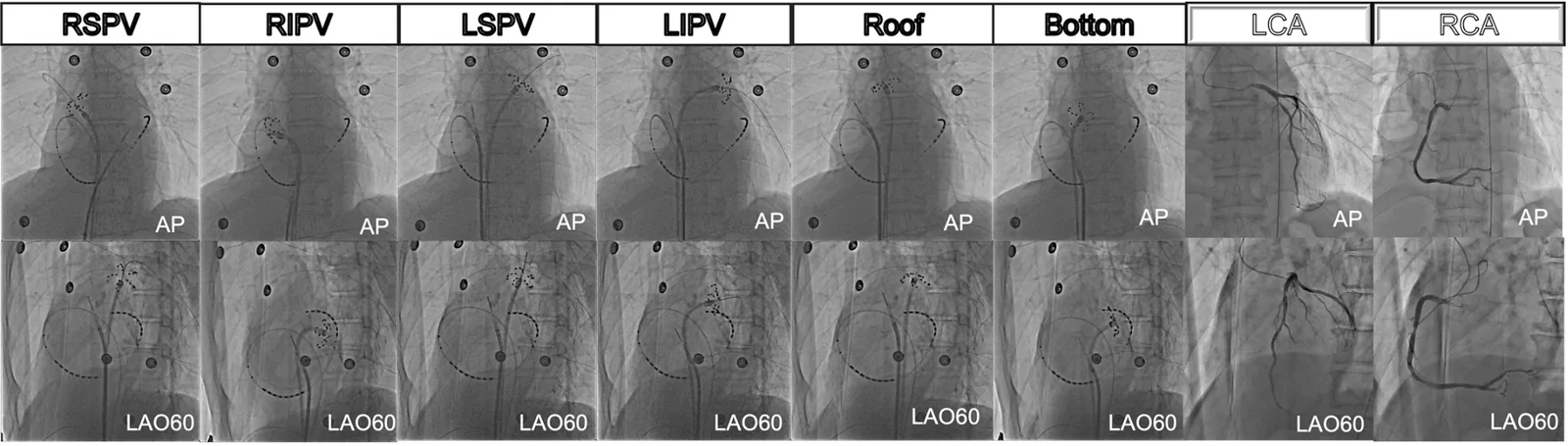
Fluoroscopic Images During PVI and PWI and Coronary Angiography Performed After PFA The upper panels show anteroposterior views, and the lower panels show left anterior oblique views at 60°. Comparison of the PFA fluoroscopic images with the coronary angiographic images in relation to the cardiac silhouette demonstrates that PFA was not delivered in areas adjacent to the coronary arteries. The panel labels denote the right superior pulmonary vein (RSPV), right inferior pulmonary vein (RIPV), left superior pulmonary vein (LSPV), left inferior pulmonary vein (LIPV), roof line, and bottom line. LCA = left coronary artery; PFA = pulsed field ablation; PVI = pulmonary vein isolation; PWI = posterior wall isolation; RCA = right coronary artery.

**Figure 4:**
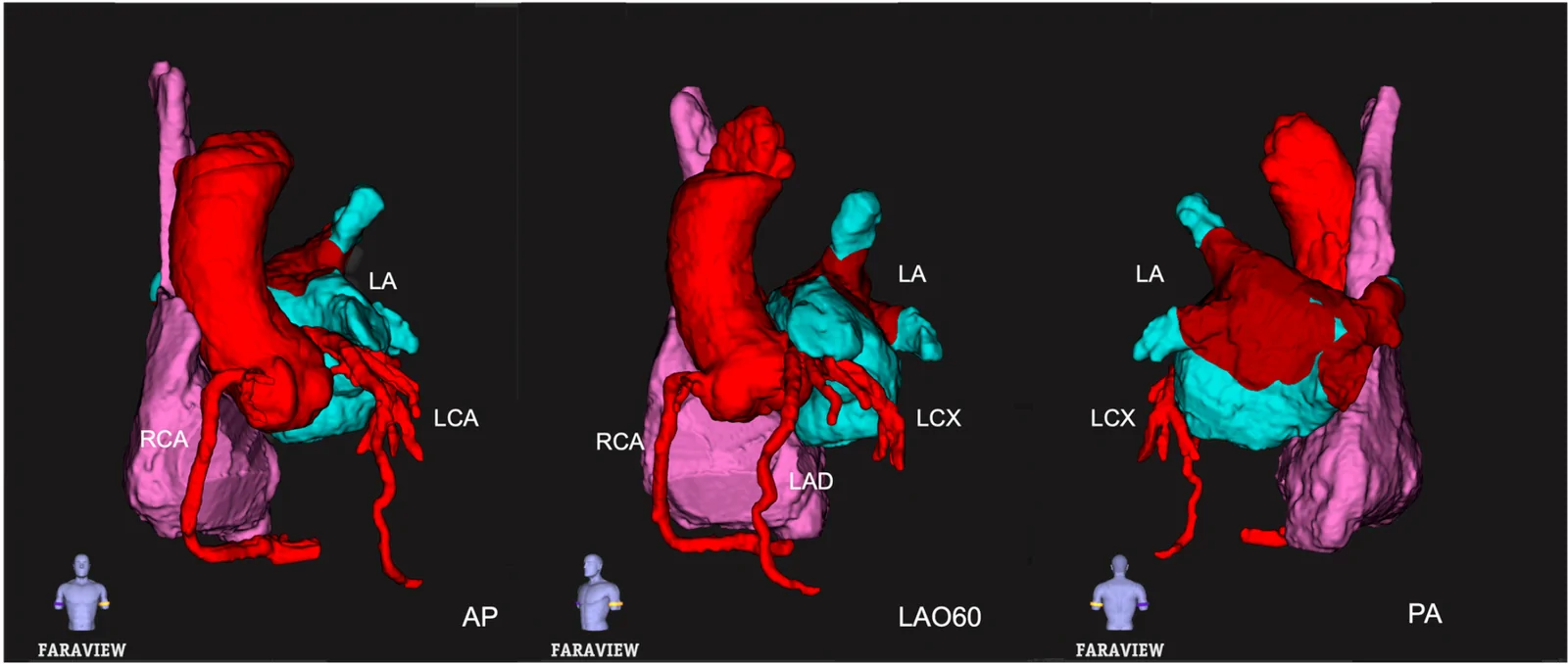
Coronary Arteries and PFA Sites on CT/FARAVIEW The coronary arteries and left atrium (LA) are shown on computed tomography (CT). The aorta and coronary arteries are colored red, the LA light blue, and the superior vena cava and right atrium pink. Labels indicate the LA, left coronary artery (LCA), left anterior descending artery (LAD), left circumflex artery (LCX), and right coronary artery (RCA). Using FARAVIEW, a 3-dimensional anatomic visualization platform, with the field tag set at 10 mm, the sites treated with the 31-mm pentaspline PFA catheter (FARAPULSE) are shown in dark red. These images demonstrate anatomical separation between the PVI/PWI sites and the epicardial coronary arteries. PFA = pulsed field ablation; PVI = pulmonary vein isolation; PWI = posterior wall isolation.

## Outcome and Follow-Up

After vasodilator therapy, the patient showed steady hemodynamic recovery without recurrent ischemic deterioration. He was discharged in stable condition on medical therapy 2 days after catheter ablation.

## Discussion

This case illustrates a severe vasospastic phenotype after anatomically remote left atrial PFA. Three features support generalized multivessel coronary vasospasm rather than localized coronary irritation or direct structural injury. First, the event occurred immediately after PVI and PWI with a 31-mm pentaspline PFA catheter, whereas the treated sites were not immediately adjacent to the epicardial coronary arteries; fluoroscopic and CT/FARAVIEW assessment supported anatomical separation between the ablation sites and coronary arteries. Second, the ECG changes were highly dynamic, with initial inferior ST-segment elevation followed by improvement in the inferior leads and new anterior ST-segment elevation. Third, diffuse narrowing in both coronary systems resolved promptly after intracoronary nitroglycerin.

Localized PFA-associated coronary vasospasm is most readily explained when energy is delivered close to a coronary artery, such as during cavotricuspid isthmus or mitral isthmus ablation. Prior reports showed that coronary-adjacent PFA can provoke severe but nitrate-responsive vasospasm, and that prophylactic or therapeutic nitroglycerin can attenuate this response.^1^, ^2^, ^3^ This proximity-based mechanism is less satisfactory in the present case because the fluoroscopic and CT/FARAVIEW findings did not support simple catheter-coronary apposition. Schaack et al^4^ reported right coronary vasospasm with ST-segment elevation and atrioventricular block despite a remote distance from the ablation site, and Mannion et al^5^ described remote diffuse multicoronary spasm after PVI and PWI using a focal monopolar PFA catheter.^4^^,^^5^ Our case extends these observations by demonstrating diffuse bilateral coronary vasospasm with cardiogenic shock after pentaspline left atrial PFA, despite imaging findings suggesting anatomical separation between the ablation sites and the epicardial coronary arteries.

The immediate onset and evolving ECG distribution are also important. A fixed obstructive event, coronary embolism, or direct thermal injury would be expected to produce a more territorially consistent pattern. In contrast, shifting ST-segment elevation together with nitrate-responsive diffuse angiographic narrowing is more consistent with dynamic vasomotor instability affecting multiple epicardial vessels. This distinction is clinically relevant because the therapeutic priority is rapid recognition and vasodilator administration rather than assuming a focal lesion requiring revascularization.

The mechanism was likely multifactorial and cannot be proven from a single case. Patients in Japanese and East Asian cohorts may have higher background susceptibility to vasospastic phenomena, and a Japanese multicenter series of atrial fibrillation ablation reported coronary artery spasm in 42 of 22,232 procedures (0.19%), most often presenting with inferior ST-segment elevation and resolving after nitrate administration.^6^ Against this substrate, left atrial ablation may perturb autonomic tone through ganglionated plexi or adjacent neural structures; multivessel coronary spasm has been described during ganglionated plexi stimulation with radiofrequency pulmonary vein isolation.^7^ PFA-specific effects may also contribute. Electroporation-related vascular or autonomic stimulation could theoretically occur beyond simple tissue proximity, and PFA-related hemolysis can release cell-free hemoglobin, which may scavenge nitric oxide and favor vasoconstriction.^8^^,^^9^ Dexmedetomidine may have further modified vasoreactivity through peripheral alpha-2B receptor-mediated vasoconstrictive effects.^10^ These possibilities should be regarded as complementary rather than mutually exclusive.

Whether this phenotype is unique to PFA remains uncertain. Thermal ablation technologies have also been associated with coronary vasospasm, so the present case should not be interpreted as proof that PFA alone causes generalized vasospasm. However, the occurrence of severe nitrate-responsive spasm after anatomically remote PFA suggests that electroporation-mediated vascular or autonomic effects may represent an additional mechanism beyond direct local tissue proximity. Further accumulation of cases with detailed spatial reconstruction, drug exposure, hemolysis markers, and coronary angiographic timing will be needed to clarify causality.

From a practical perspective, severe hypotension with ischemic ST-segment changes after PFA should trigger immediate consideration of coronary vasospasm in addition to tamponade, embolism, and acute coronary syndrome. Rapid access to coronary angiography in the electrophysiology laboratory environment is important. Intracoronary nitroglycerin has both diagnostic and therapeutic value because it can demonstrate rapid reversibility of epicardial narrowing while directly treating the affected vessel. In hemodynamically unstable patients, small intracoronary boluses titrated to angiographic response and blood pressure may be preferable to empiric high-dose systemic nitrate administration, with transition to intravenous or continuous vasodilator therapy, such as nicorandil or calcium-channel blockade, after stabilization. In the present case, immediate intracoronary nitroglycerin followed by continuous nicorandil was associated with rapid angiographic and hemodynamic recovery.

## Conclusions

Severe but reversible multivessel coronary vasospasm can occur after anatomically remote left atrial PFA for atrial fibrillation. Coronary vasospasm should be considered when hypotension or evolving ischemic ST-segment changes develop after PFA, even when ablation sites are anatomically remote from the coronary arteries.

**Equipment List:** Multivessel Coronary Vasospasm Causing Cardiogenic Shock After Anatomically Remote Pulsed Field Ablation for Atrial Fibrillation

*Pulsed field ablation catheter:* A 31-mm pentaspline pulsed field ablation catheter (FARAPULSE; Boston Scientific) for pulmonary vein isolation and posterior wall isolation.
*Mapping/visualization platform:* FARAVIEW, a 3-dimensional anatomic visualization platform used to assess the spatial relationship between ablation sites and the coronary arteries; field tag size was set at 10 mm.
*Postprocedural anatomic assessment:* Fluoroscopic comparison between ablation views and coronary angiographic views, supplemented by CT-FARAVIEW reconstruction.
*Coronary evaluation:* Emergency coronary angiography with intracoronary nitroglycerin to confirm reversible multivessel coronary vasospasm.
*Hemodynamic and respiratorysupport:* Invasive arterial pressure monitoring, noninvasive positive pressure ventilation, norepinephrine support, and systemic heparinization during the procedure.
*Sedation:* Deep sedation with dexmedetomidine and intermittent propofol.

This equipment list is provided for *JACC*: Case Reports submission and is intended to support reproducibility of the procedure and the anatomic assessment used in this case.

## Funding Support and Author Disclosures

The authors have reported that they have no relationships relevant to the contents of this paper to disclose.
